# Effects of laccase and lactic acid bacteria on the fermentation quality, nutrient composition, enzymatic hydrolysis, and bacterial community of alfalfa silage

**DOI:** 10.3389/fmicb.2022.1035942

**Published:** 2022-10-06

**Authors:** Xueyan Bao, Haoran Feng, Gang Guo, Wenjie Huo, Qinghong Li, Qingfang Xu, Qiang Liu, Cong Wang, Lei Chen

**Affiliations:** ^1^College of Animal Science, Shanxi Agricultural University, Taigu, Shanxi, China; ^2^College of Grassland Science, Shanxi Agricultural University, Taigu, Shanxi, China

**Keywords:** alfalfa silage, bio-additives, bacterial community, fermentation quality, enzymatic hydrolysis

## Abstract

Ensiling has long been as a mainstream technology of preserving forage for ruminant production. This study investigated the effects of bioaugmented ensiling with laccase and *Pediococcus pentosaceus* on the fermentation quality, nutritive value, enzymatic hydrolysis, and bacterial community of alfalfa. The application of laccase and *Pediococcus pentosaceus* combination was more potent in modulating the fermentation quality of silage than laccase and *Pediococcus pentosaceus* alone, as indicated by higher lactic acid contents and lactic acid to acetic acid ratios, and lower pH, dry matter losses, and ammonia nitrogen contents. Moreover, treatments with additive enhanced protein preservation and structural carbohydrate degradation, while increasing true protein and water-soluble carbohydrate contents. By promoting lignin degradation, treatments containing laccase further facilitated the release of sugars from cellulose compared with treatment with *Pediococcus pentosaceus* alone. The additive treatments reduced the bacterial diversity and optimized the bacterial community composition of silage, with an increase in the relative abundance of desirable *Lactobacillus* and a decrease in the relative abundance of undesirable *Enterobacter* and *Klebsiella*. PICRUSt functional prediction based on Kyoto Encyclopedia of Genes and Genomes (KEGG) databases revealed that PL and LPL treatments increased the metabolism of membrane transport, carbohydrate, and terpenoids and polyketides related to fermentation activities. It can be concluded that bioaugmented ensiling with laccase and *Pediococcus pentosaceus* combination can be an effective and practical strategy to improve silage fermentation and nutrient preservation of alfalfa silage.

## Introduction

Alfalfa is a high-yielding and nutrient-rich forage legume and is widely used as a dietary component of ruminants. The utilization of alfalfa in ruminant production can expand the protein source and thus reduce the dependency on expensive protein supplement (Tabacco et al., 2006). In many regions of the world, ensiling rather than haying is a more practical strategy for preserving forage, considering greater harvest speed and less weather damage in practice (Broderick et al., 2017). However, natural ensiling produces high-quality alfalfa silage is difficult due to the high buffering capacity and low water-soluble carbohydrate (WSC) content and attached lactic acid bacteria (LAB) number (McDonald et al., 1991). Therefore, silage additives, including chemicals (sugars and organic acid) and biologicals (LAB and enzymes), have been applied to alfalfa at ensiling to modulate the resultant silage quality. Compared with chemical additives, biological additives are more applicable in silage preparation because of cost-effective and eco-friendly properties.

Laccases, one group of multicopper-containing oxidases, are mainly present in fungi, bacteria, insects, and plants (Fillat et al., 2017). Laccases can catalyze the oxidation of both the lignin-related compounds and aromatic compounds, at the expense of molecular oxygen (Nazar et al., 2022). In the past decade, laccases have received extensive attention due to their high-efficiency applications in various industries (Debnath and Saha, 2020). Fungal laccases have been shown to effectively degrade lignin in grasses and crop residues (Rajak and Banerjee, 2018; Sherpa et al., 2018). Moreover, Chen et al. (2012) reported that fungal laccase enhanced the lignin degradation of corn stover silage compared with raw corn stover, leading to increased downstream cellulose hydrolysis. These authors ascribed the increased lignin degradation to the fact that ensiling provided access for laccase to enter the complex biomass for delignification by partially hydrolyzing cellulose and hemicellulose into soluble sugars. Therefore, it was hypothesized that the addition of fungal laccase at ensiling could degrade lignin in alfalfa and thus increase the conversion of fiber into soluble sugars for LAB growth. Besides, given that the reaction of laccase degrading lignin consumes oxygen, laccase addition may favorably create anaerobic conditions in silage and reduce the respiration losses. To our knowledge, there are little information about effects of fungal laccase on the fermentation quality and bacterial community of alfalfa silage. Furthermore, *Pediococcus pentosaceus* has been proven to be an efficient silage inoculant, because they can promote lactic acid fermentation in silage and improve nutrient preservation (Irawan et al., 2021). Accordingly, it is expected that alfalfa silage quality can be greatly improved by the addition of laccase and *P. pentosaceus* combination in consideration that they may act synergistically or additively on enhancing lactic acid fermentation and cell wall degradation.

Understanding the bacterial community composition of silage can provide deep insight into the relationship between key taxa and fermentation parameters in silage (Bai et al., 2022). Thus, extensive attempts to profile bacterial community related to silage fermentation are needed to reveal important taxa that are favorable to improving silage quality. Therefore, the objective of this study was to investigate the potential of laccase and *P. pentosaceus* bioaugmentation on modulating the performance of alfalfa silage, with special accent in the fermentation characteristics, nutrient composition, enzymatic hydrolysis, and bacterial community.

## Materials and methods

### Forage and silage preparation

Whole-plant alfalfa (cultivar SR4030) was mowed at the early flowering stage at the field of Shanxi Agricultural University. Alfalfa was field-wilted (26–31°C) for 4 h to 35% DM and chopped to about 2-cm in length using a forage harvester. The fresh forage was randomly divided into 12 piles (2 kg each pile), which were assigned to one the following treatments in triplicate: deionized water (Control), laccase (100,000 U/g, Xiasheng Industrial Group Co., Ltd., Beijing, China) at application rate of 0.04% of fresh forage (LA), *P. pentosaceus* 3XM4512 (GenBank accession number MF623192.1) to achieve 1 × 10^6^ cfu/*g* of fresh forage (PL), and a combination laccase and *Pediococcus pentosaceus* (LPL), respectively. The strain of *P. pentosaceus* was inoculated in De Man, Rogosa, and Sharpe (MRS) broth (Huankai Biotechnology, Co., Ltd., Guangdong, China) at 37°C for 20 h, and then centrifuged at 3,000 *g* for 20 min to obtain the bacterial pellet. The pellet was washed three times with sterile NaCl (0.9%, *w*/*v*), and resuspended in sterile deionized water to be a concentration of 10^9^ cfu/ml (measured by OD at 600 nm). All additives were diluted in sterile deionized water to obtain the target application rate and sprayed uniformly onto the forages at a rate of 10 ml/kg. Approximately 500 g of raw material was placed into a vacuum-packed bag silo (25 width × 36 cm height) and heat sealed with a vacuum packaging machine (YMX-958-6l, Yiminxin Co., Ltd., Quanzhou, China). The experimental silos were weighed and stored at room temperature (22–25°C) for 90 days. The bags were weighed again on opening to calculate DM losses (DML) of silage due to fermentation. Once silos were opened, subsamples were prepared for the determination of fermentation end-products, enzymatic hydrolysis, and bacterial community. Fresh alfalfa subsamples from the initial pile of chopped alfalfa were collected for chemical and microbial analyses.

### Chemical and microbial analyses

The first subsamples (100 g) were oven-dried at 55°C for 72 h, and then ground passing through a 1-mm screen (Ke et al., 2017). The contents of DM (method 930.15), acid detergent fiber (ADF, method 973.18), and crude protein (CP, method 984.13) were analyzed according to the Association of Official Analytical Chemists (AOAC, 1990). The protein fractions including non-protein nitrogen (PA) and true protein (PB) were determined with the method of Licitra et al. (1996). For the neutral detergent fiber (NDF) and acid detergent lignin (ADL) analyses, ground materials were treated with heat-stable α-amylase and sodium sulfite as described by Van Soest et al. (1991). Hemicellulose (HC) and cellulose (CL) were estimated as the differences between NDF and ADF and the differences between ADF and ADL, respectively. The WSC was determined by anthrone sulfuric acid calorimetry method according to Zhao et al. (2021).

The second subsamples (20 g) were mixed with 60 ml of deionized water and left to stand at 4°C for 24 h to prepare water extracts (Chen et al., 2021). The water extracts were filtered through Whatman filter papers, and the filtrates were used to determine pH, ammonia nitrogen (NH_3_–N) and free amino acid nitrogen (AA–N), organic acids (lactic, acetic, propionic, and butyric acids) and ethanol. The pH was immediately measured using an electrode pH meter (P901, YOKE Instrument Co., Ltd., Shanghai, China). The NH_3_–N and AA–N were determined according to Broderick and Kang (1980). The organic acids and ethanol were analyzed using HPLC (1,260, Agilent Technologies, Inc., Waldbronn, Germany) coupled with a refractive index detector, on an Carbomix^®^ H-NP5 column (Sepax Technologies, Inc., Newark, DE, United States), following the parameters: mobile phase, 2.5 mmol/l H_2_SO_4_; flow rate, 0.5 ml/min and oven temperature, 55°C. The third subsample (10 g) was blended with sterilized saline solution (NaCl, 9.0 g/kg) at a ratio of 1:9 *w*/*v*, homogenized for 20 min at 25°C in a table shaker (120 rpm/min). Ten-fold serial dilutions were prepared, and LAB and yeast numbers were analyzed with the pour plate method (Chen et al., 2021). In brief, the numbers of LAB were enumerated on de Man, Rogosa, Sharpe (MRS) agar (Huankai Biotechnology, Co., Ltd., Guangdong, China) incubated at 30°C for 48 h; and that of yeasts was enumerated on malt extract agar (Huankai Biotechnology, Co., Ltd., Guangdong, China) incubated at 32°C for 72 h.

### Enzymatic hydrolysis of alfalfa silage

The hydrolysis of fresh forage and alfalfa silage was carried out in a 100 ml Erlenmeyer flask containing 10% *w*/*w* substrate and 0.1 M sodium citrate buffer (pH 4.8). The hydrolysis enzymes were added into the mixture at a dose of cellulase 10 FPU/g and β-glucosidase 10 CBU/g per gram of cellulose (Travaini et al., 2013). The cellulase and β-glucosidase were purchased from Solarbio Science & Technology Co., Ltd. (Beijing, China). For preventing microbial contamination, agent sodium azide (0.1% *w*/*v*) was applied to the reaction mixture of hydrolysis. Hydrolysis was performed at 50°C for 48 h in a shaking inoculator (150 rpm). Samples from hydrolysates were collected and centrifuged at 10, 000 × *g* for 10 min. The supernatants were analyzed for reducing sugar yield with the dinitrosalicylicacid method (Miller, 1959).

### Bacterial community composition

Bacterial community analysis of silages in each treatment was performed using 16S rDNA sequencing technology at Lianchuan Biotechnology Co., Ltd. (Hangzhou, China). Genomic DNA of bacteria from silage samples was extracted using Tiangen DNA extraction kit (DP 705, Tiangen Bitech, Co., Ltd., Beijing, China). The V3–V4 regions of 16S rDNA gene was amplified using primers 341F: (5′-CCTACGGGNGGCWGCAG-3′) and 805R: (5′-GGACTACHVGGGTATCTAAT-3′), which were tagged with specific barcode at the 5′ ends of the primers (Logue et al., 2016). Each PCR amplification was run with a final 25 μl reaction mixture containing 12.5 μl PCR Premix, 2.5 μl of each primer, 25 ng of template DNA, and PCR-grade water to adjust the volume. The procedure of PCR amplification and gel electrophoresis for checking amplicons were described in Du et al. (2019). The amplicons were purified by AMPure XT beads (Beckman Coulter Genomics, Danvers, MA, United States) and quantified by Qubit (Invitrogen, United States). The purified amplicons were pooled in equimolar proportions, and then paired-end sequenced using the Illumina NovaSeq PE250 platform in by LC-Bio Technology Co., Ltd. (Zhejiang, China). Paired-end reads were assigned to samples according to the unique barcodes, trimmed by truncating the barcodes and primer sequences, and merged using FLASH. The feature table and feature sequence were obtained by screening and quality filtration with fqtrim (v0.94), chimera removal using Vsearch software (v2.3.4), and chimeric sequence dereplication and filtering using DADA2. Subsequently, the obtained sequences were assigned into amplicon sequence variants (ASVs), which were used for species annotation in the SILVA database at a confidence cut-off of 0.7. Alpha diversity and beta diversity were calculated by normalized to the same random sequences. Feature abundances were then normalized using the relative abundance of each sample according to the SILVA (release 132) classifier. Alpha diversity indices including Shannon, Simpson, Chao1, and Good’s coverage were applied in analyzing complexity of species diversity for samples. Beta diversity was calculated by principal coordinate analysis (PCoA) based on UniFrac metrics, and statistical comparisons amongst groups were conducted using ANOSIM. Alpha diversity and Beta diversity were all calculated by QIIME2 script. Functional gene prediction based on Kyoto Encyclopedia of Genes and Genomes (KEGG) databases was conducted using PICRUSt2 according to Langille et al. (2013). Other diagrams were implemented using the R packages. Sequence data were deposited in NCBI’s Sequence Read Archive under BioProject accession number PRJNA875211.

### Statistical analysis

Data for microbial numbers were transformed by log_10_ and presented on a fresh matter (FM) basis. All statistical analyses were performed using the variance (ANOVA) by the GLM procedure of SAS 9.2 (SAS Institute Inc.; Cary, NC, USA). The model for data analysis was: *Y_i_* = *μ* + *α_i_* + *ε_i_*; where *Y_i_* is the dependent variable, *μ* is the general mean, *α_i_* is the treatment effect, and *ε_i_* is the experimental error. The treatment differences between least square means were determined using the Tukey’s test, and significance was declared at *p* < 0.05.

## Results

### Initial characteristics of fresh alfalfa

The chemical composition and microbial numbers of fresh alfalfa are shown in Table 1. The contents of DM and WSC in alfalfa were 357 g/kg FM and 49.2 g/kg DM, respectively. Alfalfa contained CP, NDF, ADF, and lignin of 225, 406, 329, and 66.7 g/kg DM, respectively. The LAB numbers of alfalfa silage were 4.56 log_10_ cfu/g FM.

**Table 1:** Chemical composition and microbial counts of fresh alfalfa.

Item	Alfalfa
Dry matter (g/kg FM)	357
pH	6.19
CP (g/kg DM)	225
NDF (g/kg DM)	406
ADF (g/kg DM)	329
ADL (g/kg DM)	66.7
Hemicellulose (g/kg DM)	77.3
Cellulose (g/kg DM)	263
WSC (g/kg DM)	49.2
LAB (log_10_ cfu/g FM)	4.16
Yeasts (log_10_ cfu/g FM)	4.62

FM, fresh matter; CP, crude protein; NDF, neutral detergent fiber; ADF, acid detergent fiber; ADL, acid detergent lignin; WSC, water-soluble carbohydrate; LAB, lactic acid bacteria.

### Fermentation characteristics of alfalfa silage

The fermentation parameters of alfalfa silages were different among treatments (Table 2). All additive treatments deceased (*p* < 0.05) the pH, acetic acid, butyric acid, ethanol, NH_3_–N, and AA–N contents, and DML relative to the CK treatment, while increased (*p* < 0.05) lactic acid contents and LAB numbers. The LPL treatments produced the largest decrease in pH, acetic acid, ethanol, NH_3_–N, and AA–N contents, and DML, and produced the largest increase in lactic acid content.

**Table 2:** Fermentation characteristics and losses of alfalfa silage treated without or with different additives.

Item	Treatments				SEM	Value of *p*
	CK	LA	PL	LPL		
pH	5.05^a^	4.69^b^	4.53^c^	4.41^d^	0.07	<0.001
Lactic acid (g/kg DM)	43.9^d^	58.8^c^	65.6^b^	75.7^a^	3.49	<0.001
Acetic acid (g/kg DM)	38.6^a^	26.3^b^	18.5^c^	18.2^d^	2.50	<0.001
Lactic:acetic acid	1.14^d^	2.24^c^	3.55^b^	4.15^a^	0.35	<0.001
Propionic acid (g/kg DM)	0.31	ND	ND	ND	–	–
Butyric acid (g/kg DM)	1.97^a^	1.04^b^	0.55^c^	0.29^d^	0.19	<0.0001
Ethanol (g/kg DM)	6.01^a^	3.86^b^	3.20^c^	2.44^d^	0.40	<0.001
NH_3_-N (g/kg TN)	125.2^a^	89.5^b^	77.8^c^	65.1^d^	6.77	<0.001
AA-N (g/kg TN)	392^a^	364^b^	346^c^	325^d^	7.50	<0.0001
DML (g/kg)	67.5^a^	56.8^b^	45.2^c^	33.9^d^	3.81	<0.001
LAB (log_10_ cfu/g FM)	7.88^b^	8.21^a^	8.19^a^	8.26^a^	0.05	<0.001
Yeasts (log_10_ cfu/g FM)	ND	ND	ND	ND	–	–

NH_3_–N, ammonia nitrogen; AA–N, free amino acid nitrogen; DML, dry matter losses; LAB, lactic acid bacteria; ND, not detected.

CK, no additives; LA, laccase; PL, *Pediococcus pentosaceus*; LPL, laccase + *Pediococcus pentosaceus*.

Within a row, means without a common superscript letter (a–d) differ (*p* < 0.05).

### Nutrient composition and enzymatic hydrolysis of alfalfa silage

The addition of additives resulted in a higher (*p* < 0.05) DM content than the CK, but no differences in DM content were observed among the additive treatments (Table 3). Although silage CP was unaffected by the additive treatments, protein fraction (PA and PB) contents were clearly affected by the additive treatments. The additive-treated silages had a lower (*p* < 0.05) content of PA and a higher (*p* < 0.05) content of PB than the CK silage, and LPL silage had the lowest PA content and the highest PB content. In addition, LA, PL, and LPL silages had lower (*p* < 0.05) contents of NDF, ADF, hemicellulose, and cellulose than the CK silage, and these variables were lowest in LPL silage. The ADL was reduced (*p* < 0.05) by LA and LPL treatments. The WSC content was higher (*p* < 0.05) in silage treated with PL and LPL compared with the CK silage, but was even higher (*p* < 0.05) in silage treated with LA.

**Table 3:** The nutrient composition of alfalfa silage treated without or with different additives.

Item	Treatments				SEM	Value of *p*
	CK	LA	PL	LPL		
DM (g/kg FM)	353	363	361	364	1.80	0.074
CP (g/kg DM)	219	225	228	230	1.66	0.114
PA (g/kg CP)	631^a^	604^b^	581^c^	557^d^	8.32	<0.001
PB (g/kg CP)	276^d^	299^c^	320^b^	346^a^	7.88	<0.001
NDF (g/kg DM)	407^a^	365^c^	384^b^	362^c^	5.42	<0.001
ADF (g/kg DM)	341^a^	316^c^	329^b^	310^d^	3.62	<0.001
ADL (g/kg DM)	68.9^a^	57.5^b^	67.2^a^	54.8^b^	1.85	<0.001
Hemicellulose (g/kg DM)	65.7^a^	49.4^c^	55.6^b^	51.4^c^	1.93	<0.001
Cellulose (g/kg DM)	272^a^	258^bc^	262^b^	256^c^	1.98	<0.001
WSC (g/kg DM)	9.53^d^	20.6^a^	11.9^c^	18.3^b^	1.37	<0.001

FM, fresh matter; CP, crude protein; PA, non-protein nitrogen; PB, true protein; NDF, natural detergent fiber; ADF, acid detergent fiber; ADL, acid detergent lignin; WSC, water-soluble carbohydrate.

CK, no additives; LA, laccase; PL, *Pediococcus pentosaceus*; LPL, laccase + *Pediococcus pentosaceus*.

Within a row, means without a common superscript letter (a–d) differ (*p* < 0.05).

**Figure 1:**
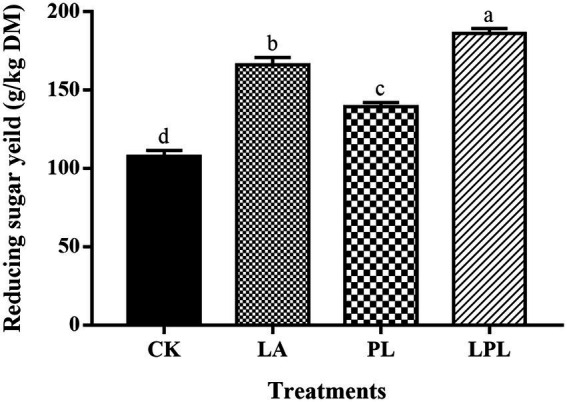
Reducing sugar yield of alfalfa silage treated with or without additives after enzymatic hydrolysis. CK, no additives; LA, laccase; PL, *Pediococcus pentosaceus*; LPL, laccase + *Pediococcus pentosaceus*. Different letters (a–d) above the column were significant (*p* < 0.05).

The enzymatic reducing sugar yield of alfalfa silage was shown in Figure 1. The LA, PL and LPL treatments further enhanced (*p* < 0.05) the reducing sugar production compared with the CK treatment, and LPL treatment resulted in the highest reducing sugar yield.

### Bacterial community of alfalfa silage

**Figure 2:**
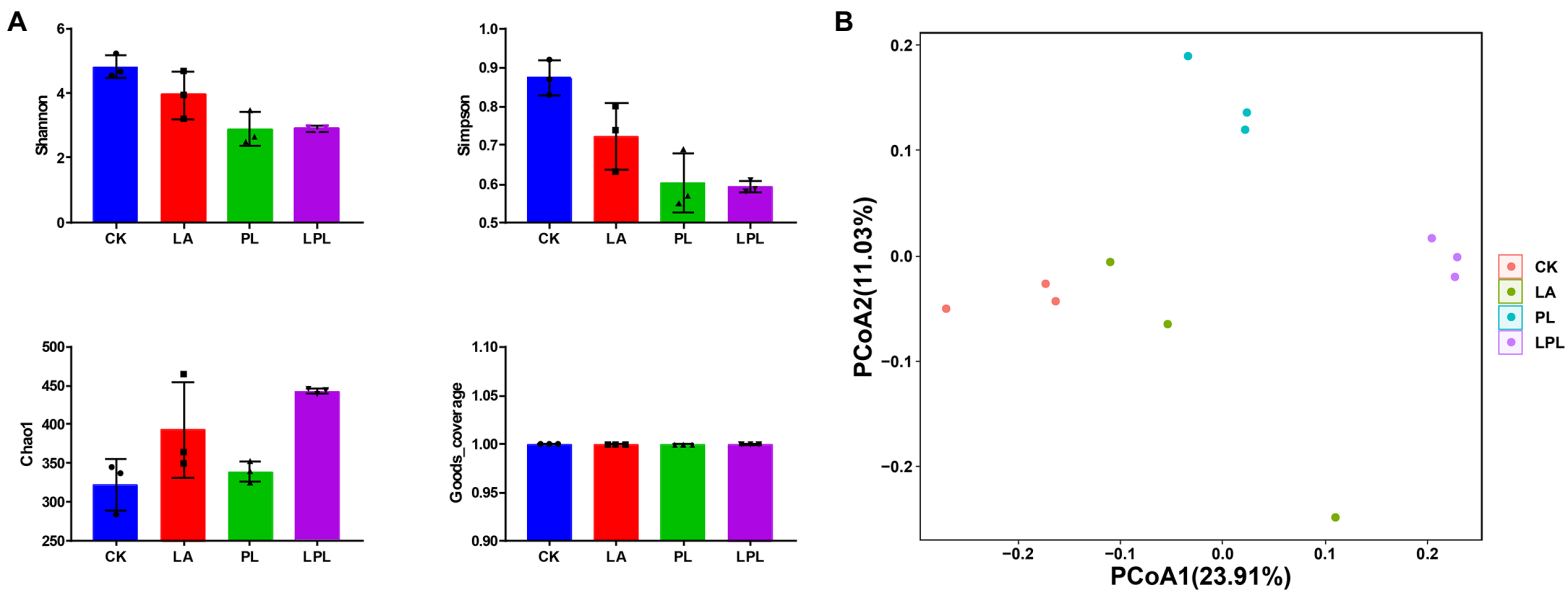
Bacterial community diversities of alfalfa silage treated with or without additives. **(A)** Alpha-diversity of bacterial community (Shannon, Simpson, Chao1, and Goods_coverage). **(B)** Principal coordinates analysis plots with unweighted Unifrac dissimilarity of bacterial community. ANOSIM value, *R*-squared: 0.75, *p* = 0.001. CK, no additives; LA, laccase; PL, *Pediococcus pentosaceus*; LPL, laccase + *Pediococcus pentosaceus*.

In total, 1,012,847 DNA sequences were detected in the 12 silage samples, and 869,281 clean DNA sequences were obtained by the quality-filtering and chimera-removal steps. Goods_coverage value in all silage samples was 1.00 (Figure 2A). Compared with the CK treatment, the additive treatments decreased Shannon and Simpson values and increased Chao1 values. The PCoA plot for sequence similarities using the unweighted UniFrac displayed a clear clustering of the bacterial community by different treatments (Figure 2B). Moreover, the ANOSIM test (*R* = 0.75, *p* = 0.001) revealed that the between-treatment variation outweighed the within-treatment variation.

**Figure 3:**
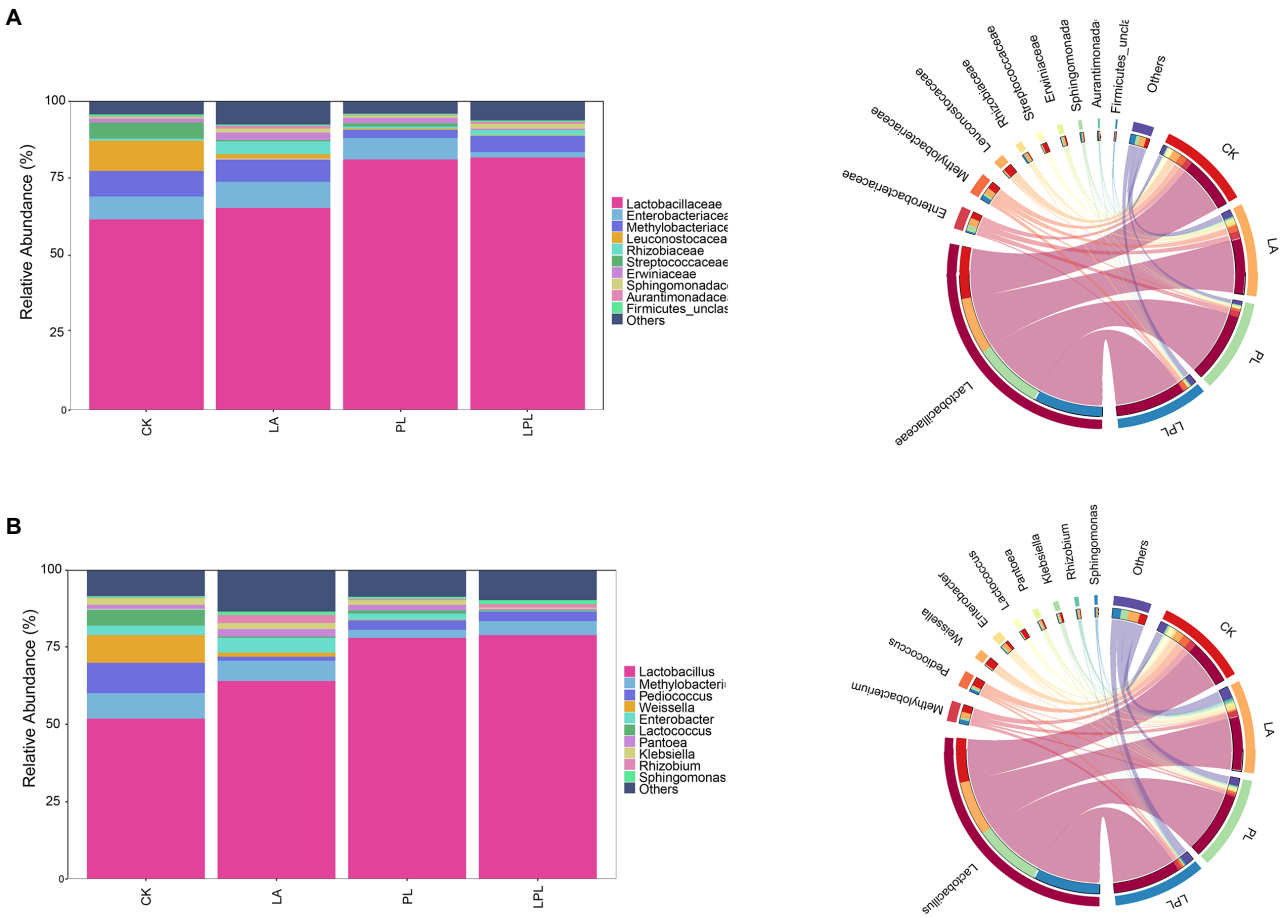
Relative abundance of bacterial community at family level **(A)** and at genus level **(B)** in alfalfa silage treated with or without additives. CK, no additives; LA, laccase; PL, *Pediococcus pentosaceus*; LPL, laccase + *Pediococcus pentosaceus*.

Generally, *Lactobacillaceae*, *Enterobacteriaceae* and *Methylobacteriaceae* were the main families in all silages (Figure 3A). Specifically, the relative abundance (RA) of *Lactobacillaceae*, *Enterobacteriaceae* and *Methylobacteriaceae* averaged 72.5, 6.14% and 5.90, respectively. The additive treatments increased the RA of *Lactobacillaceae* and decreased the RA of *Methylobacteriaceae*. The *Lactobacillaceae* abundance in the LPL silage was highest, followed by that of PL and LA silages. Moreover, the RA of *Enterobacteriaceae* was extensively lowered by LPL treatment compared with the other treatments. The CK silage had a high RA of *Leuconostocaceae* and *Streptococcaceae*. Figure 3B shows the most abundant genera using the taxonomic classification of the microbiota. Compared to the CK treatment, the additive treatments increased the RA of *Lactobacillus* and decreased the RA of *Pediococcus*, *Weissella*, and Lactococcus. The LA, PL, and LPL treatments decreased the RA of *Methylobacterium* and *Klebsiella*, while increased the RA of *Rhizobium* and *Sphingomonas*. Moreover, the RA of *Enterobacter* was reduced by PL and LPL treatments, but was increased by LA treatment compared with the CK treatment.

### Correlation analysis between bacterial community and fermentation parameters

**Figure 4:**
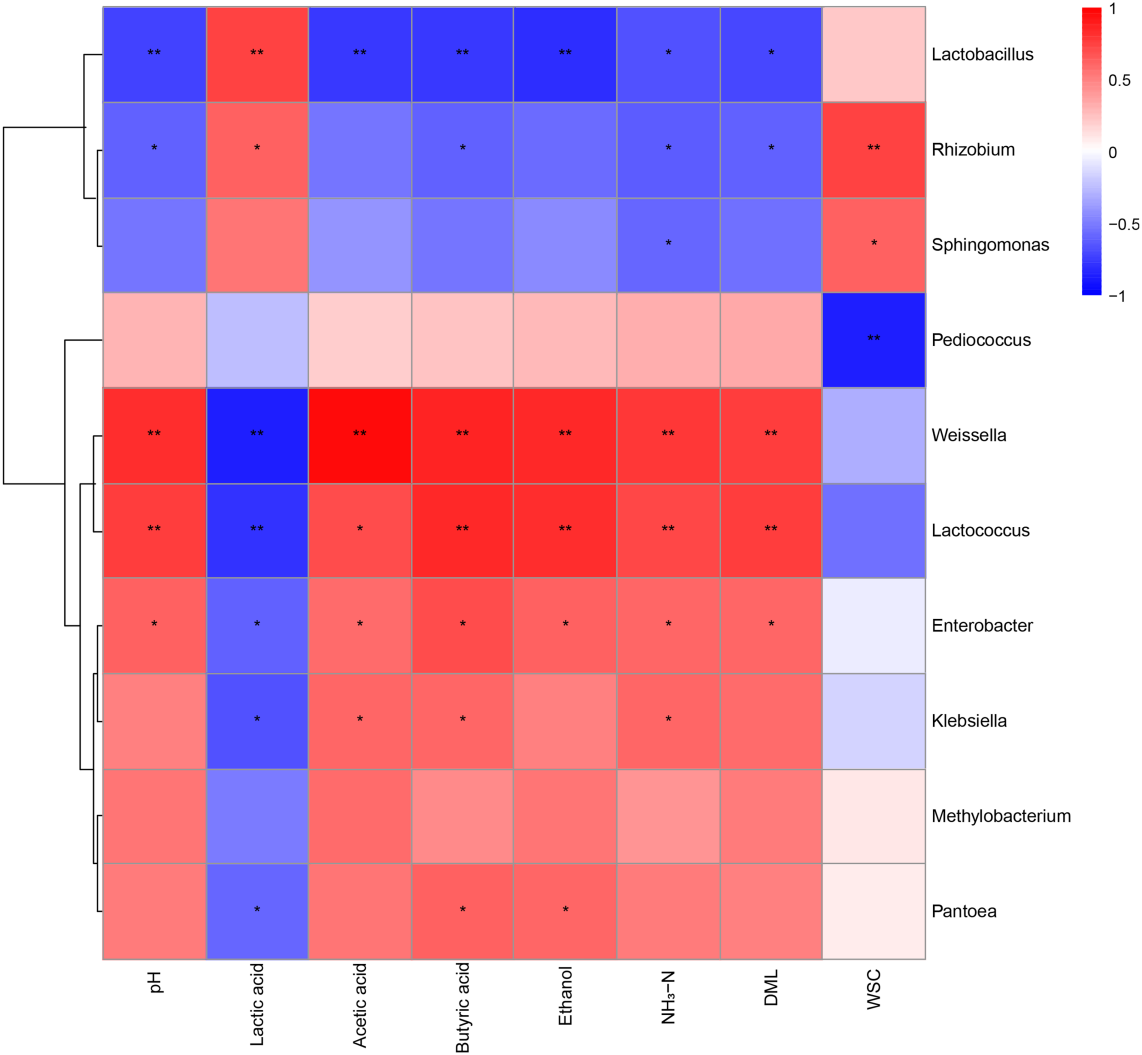
Heatmap of Spearman correlation analysis of bacterial community composition and fermentation parameters. CK, no additives; LA, laccase; PL, *Pediococcus pentosaceus*; LPL, laccase + *Pediococcus pentosaceus*. ^*^0.01 < *p* < 0.05; ^**^p < 0.01. NH_3_–N, ammonia nitrogen; DML, dry matter losses; WSC, water-soluble carbohydrate.

The pH, individual organic acids, ethanol, NH_3_-N, and WSC contents, and DML were correlated with several bacterial taxa (Figure 4). Silage pH had positive correlation (*p* < 0.05) with the genera *Weissella*, *Lactococcus*, and *Enterobacter*, but negative correlation (*p* < 0.05) with *Lactobacillus* and *Rhizobium*. Lactic acid was positively correlated (*p* < 0.05) with the genera *Lactobacilli* and *Rhizobium*. However, it was negatively correlated (*p* < 0.05) with the general *Weissella*, *Lactococcus*, *Enterobacter*, *Klebsiella*, and *Pantoea*. Acetic acid was positively correlated (*p* < 0.05) with the genera *Weissella*, *Lactococcus*, *Enterobacter* and *Klebsiella*, and negatively correlated (*p* < 0.05) with *Lactobacillus*. In addition, butyric acid was positively correlated (*p* < 0.05) with the genera *Weissella*, *Lactococcus*, *Enterobacter*, *Klebsiella*, and *Pantoea*, but negatively correlated (*p* < 0.05) with the genera *Lactobacillus* and *Rhizobium*. Ethanol had a positive correlation (*p* < 0.05) with the general *Weissella*, *Lactococcus*, *Enterobacter*, and *Pantoea*, and a negative correlation (*p* < 0.05) with the genus *Lactobacillus*. The NH_3_-N was positively correlated (*p* < 0.05) with the general *Weissella*, *Lactococcus*, *Enterobacter*, and *Klebsiella*, and negatively correlated (*p* < 0.05) with *Lactobacillus*, *Rhizobium*, and *Sphingomonas*. The DML also revealed correlations with some taxa, including positive correlation (*p* < 0.05) with the genera *Weissella*, *Lactococcus*, and *Enterobacter*, but negative correlation (*p* < 0.05) with *Lactobacillus* and *Rhizobium*.

### 16S rDNA gene-predicted functional profiles of alfalfa silage

**Figure 5:**
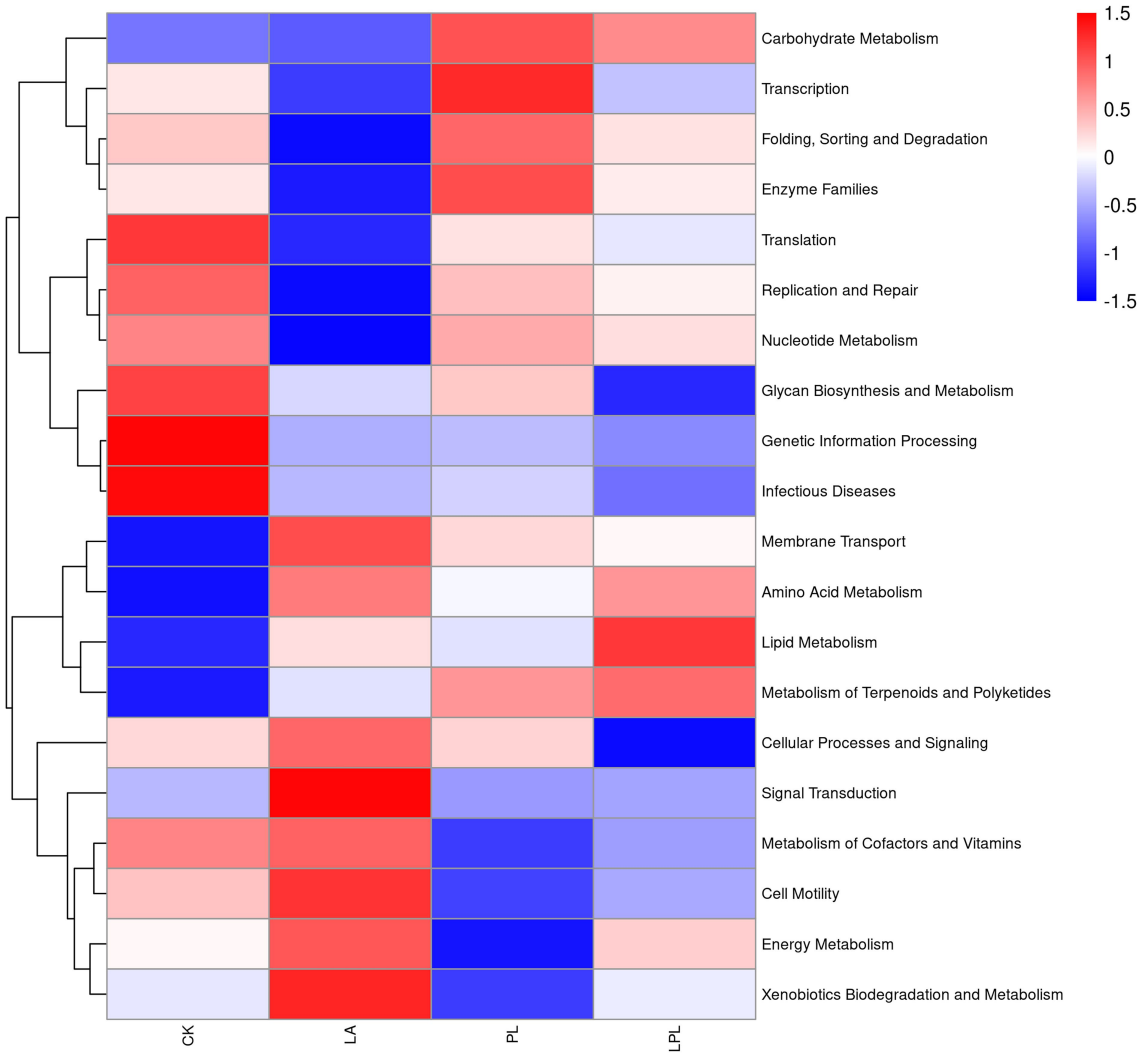
Heatmap of 16S rDNA gene-predicted functional profiles using PICRUSt. CK, no additives; LA, laccase; PL, *Pediococcus pentosaceus*; LPL, laccase + *Pediococcus pentosaceus*.

The 16S rDNA gene-predicted functions of microbiota in the CK silage and additive-treated silage are shown in Figure 5. Compared with the CK, the addition of additives upregulated the metabolism of membrane transport, amino acid, lipid, and terpenoids and polyketides, biosynthesis of other secondary metabolites, and downregulated the metabolism of replication and repair, translation, nucleotide metabolism, transcription, genetic information processing, glycan biosynthesis, and infectious diseases. The LA and LPL treatments increased the metabolism of energy and xenobiotics biodegradation. The PL and LPL enriched carbohydrate metabolism.

## Discussion

### Fermentation quality of alfalfa silage

In this study, the chemical composition of fresh alfalfa was comparable to the general values reported by Muck and Hintz (2003) and Contreras-Govea et al. (2013). The conditions for applying additives to enhance alfalfa silage fermentation were almost ideal: a slightly higher DM content (357 g DM/kg) would weaken normal fermentation, a limited WSC content, and epiphytic LAB numbers below 1 × 10^5^ cfu/g FM (McDonald et al., 1991). It is generally accepted that silage pH is a quality indicator of silage, and pH 4.30 to 5.00 is considered as the pH range for well-preserved alfalfa silage (Kung et al., 2018). All additive treatments reduced the pH of alfalfa silage compared with the CK treatment, and the pH values for the additive treatments were within that range. Due to its low pKa (3.86), lactic acid is the most critical organic acid to rapidly lower silage pH (Kung et al., 2018). Thus, the decreased pH in the additive-treated silages was most likely due to the increased lactic acid formation by the additives. Together with decreased acetic acid content and increased lactic acid to acetic acid ratio in these silages, this indicated that the additive treatments shifted the fermentation pattern toward a more homolactic fermentation. In agreement with previous studies (Filya et al., 2007; Queiroz et al., 2018), inoculation with *P. pentosaceus* led to silages with an enhanced homolactic acid fermentation and lactic acid production. Importantly, the increased lactic acid content in LA silage confirmed the hypothesis that laccases have a positive effect on the extent of lactic acid fermentation. Delignification by laccase would expose readily hydrolysable fiber fractions in silage, which were hydrolyzed by organic acids to yield more sugars for lactic acid fermentation. Another reason could be that the accelerated oxygen consumption due to delignification by laccase inhibited plant and aerobic microorganism activities, and thus saved more fermentable sugars for lactic acid fermentation. These assumptions were partly supported by decreased hemicellulose and cellulose contents as well as increased WSC content in LA silage. The highest lactic acid content in LAL silage may be due to the synergistic effect of LA and PL treatments, with sugars being efficiently used by LAB to produce lactic acid. The formation of butyric acid and ethanol during ensiling accompanies carbon dioxide production, which can cause substantial DM and energy losses in the silage (Blajman et al., 2020). Thus, lower butyric acid and ethanol contents and DML in the additive-treated silages than the CK silage indicated a reduction in fermentation loss of alfalfa silage. The reduction in these variables was due to the enhanced lactic acid production and pH decline by the additive treatments, which inhibited the metabolic activities related to butyric acid and ethanol formation (Borreani et al., 2018).

### Nutrient composition and enzymatic hydrolysis

Forage true proteins are firstly converted into peptides and free amino acids by plant enzymes, and these nitrogenous compounds are further hydrolyzed into amides, amines, and ammonia by the microbial deamination activities (Albrecht and Muck, 1991). Compared with NPN, true protein is more efficiently utilized by ruminants, thereby increasing forage protein available for digestion and absorption in the small intestines (He et al., 2021; Bachmann et al., 2022). In this study, the additive treatments decreased the NPN, NH_3_–N, and AA–N contents, and increased the true protein content relative to the CK treatment. The present results indicated that the addition of additives inhibited the proteolysis and improved the protein quality of alfalfa silage. It seemed to be a consequence of restricted plant and microbial proteolytic processes due to the accelerated pH decline by the additive treatments. Regarding to carbohydrate compositions, the LA, PL, and LPL treatments increased the degradation of hemicellulose and cellulose by 24.7 and 5.17%, 15.3 and 3.5%, and 21.7 and 6.09%, respectively, mainly accounting for the decreased NDF and ADF of silage. It should be noted that LA and LPL treatments were more effective in degrading fibers than PL treatment. This might because lignin degradation by laccase destroyed cellulose–hemicellose–lignin network structure and thus enhanced acid hydrolysis in cellulose and hemicellulose (Chen et al., 2012). Accordingly, the WSC content increased to varying degrees by the additive treatments. The higher residual WSC contents in additive-treated silages indicated not only an efficient fermentation, but also a more available fermentable substrate to ruminal microbes.

Enzymatic hydrolysis is a simple method to evaluate the carbohydrate digestion property of silage biomass in microbial fermentation (He et al., 2021). Compared with the CK treatment, a higher reducing sugar yields was observed for the additive treatments. The increased reducing sugar production was most likely due to the removal of lignin and hemicellulose as aforementioned. Sun et al. (2019) reported that the removal of hemicellulose and lignin increased the porosity in the silage biomass, thus making cellulose more accessible to cellulase and enhancing reducing sugar production. Overall, the addition of laccase and *P. pentosaceus* improved the fermentation quality and preserved more nutrients of alfalfa silage, and LA–PL combination had a beneficial synergistic effect.

### Bacterial diversity and composition of alfalfa silage

According to the Goods_coverage values (> 0.99), the sequencing analysis revealed that coverage of bacterial diversity was sufficient to represent the bacterial community composition of silage. In this study, the bacterial diversity was reduced in the additive-treated silages, especially for PL and LPL silages, compared with the CK silage. The additive treatments increased the RA of predominant genus *Lactobacillus*, thus decreasing bacterial diversity. It was commonly accepted that the diverse of microbial community is negatively related to the abundance of predominant bacteria (Polley et al., 2007; Allen et al., 2009). In the present study, the decrease in the bacterial diversity may result from the pH decline as aforementioned, which inhibited the growth of the other bacteria (Zi et al., 2021). Furthermore, PCoA indicated that the additive treatments reshaped the structure of bacterial community of silage, and reflected the turnover of species from the CK to additive-treated silages.

The main bacterial species performing lactic acid fermentation in silage generally belong to the genera *Lactobacillus*, *Pedicoccus*, and *Lactococcus* of the family *Lactobacillaceae* and to the general *Weissella*, and *Leuconostoc* of the family *Leuconostocaceae* (Pang et al., 2011; Gharechahi et al., 2017). Our study revealed that 71.4% of the bacterial community in the CK silage belong to the families *Lactobacillaceae* and *Leuconostocaceae*, comprising by the genera *Lactobacillus*, *Pediococcus*, *Lactococcus*, and *Weissella*. This was consistent with the findings of Ogunade et al. (2018) and Su et al. (2019), who reported that majority of genera detected in alfalfa silage are *Lactobacillus*, *Pediococcus*, *Lactococcus*, and *Weissella*. The additive treatments reduced the RA of *Pediococcus*, *Weissella*, and *Lactococcus* of alfalfa silage, but increased that of *Lactobacillus*. This was most likely due to the fact that the additive treatments accelerated the pH decline during ensiling and lowered final pH of silage (Ogunade et al., 2018). *Pediococcus*, *Weissella*, and *Lactococcus* are known to function as the early initiators of lactic acid fermentation, but would be gradually outcompeted by acid-tolerant *Lactobacillus* species as fermentation progresses (Cai et al., 1999). Moreover, this may explain the negative relationship between *Weissella* and *Lactococcus* and lactic acid content, because these two genera are replaced by *Lactobacillus* at low pH resulted from lactic acid accumulation. Furthermore, most species of *Weissella* convert WSC into both lactic and acetic acids *via* the heterofermentative pathway (Graf et al., 2016). Therefore, the decreased *Weissella* abundance in the additive-treated silages partly contributed to lower acetic acid contents and lactate-to-acetate ratios in these silages.

*Methylobacterium* are strictly aerobic and neutrophilic bacteria, however, *Methylobacterium* dominated in the CK silage. Similarly, *Methylobacterium* has also been found in large quantities in Sorghum-Sudangrass Hybrid silages (Dong et al., 2022). Ogunade et al. (2018) reported *Methylobacterium* had a positive correlation with silage pH. Moreover, Guo et al. (2020) reported that *Methylobacterium* was positively correlated with NH_3_–N content and negatively correlated with lactic acid content. However, Spearman correlation analysis revealed no relationship between *Methylobacterium* and fermentation parameters in alfalfa silage in this study. The species of *Enterobacteriaceae* are considered as undesirable microorganisms in silage because they are associated with DML and proteolysis (Zhao et al., 2021). Previous studies have reported that members of the genus *Enterobacter* can ferment glucose and lactic acid into acetic acid and ethanol, and degrade protein into ammonia (Pahlow et al., 2003; Borreani et al., 2018). Compared with the CK silage, the decreased RA of *Enterobacter* in the PL and LPL silages may be partly responsible for their lower acetic acid, ethanol, and NH_3_-N content. Queiroz et al. (2018) have reported that enterobacteria are susceptible to low pH in silage, surprisingly, LA treatment increased the *Enterobacter* abundance despite lower pH in the LA silage compared with the CK silage. Considering that LAB inoculants can not only reduce the pH at silo opening, but also accelerate the initial acidification rate of silage. This indicates the initial acidification in LA silage is not fast enough, thus *Enterobacter* may remain active and in high numbers for a longer time. Moreover, a lower RA of *Enterobacter* was found in LPL silage than LA and PL silages, indicating a synergistic reduction on *Enterobacter* by LA and PL. *Klebsiella* belongs to the family *Enterobacteriaceae* and is a group of acid-intolerant facultative anaerobe (Xian et al., 2022). Some strains of *Klebsiella* consume glucose producing 2,3-butanediol as the major end-products (Grimont and Grimont, 2015). Moreover, *Klebsiella* is a spoilage-associated microorganism in silage, and can cause mastitis in animals. Thus, the inhibition on the growth of *Klebsiella* by the additive treatments would favor silage fermentation. *Pantoea* is a genus separated from the genus *Enterobacter*, which is inferred to compete with lactic acid bacteria for nutrients and to cause butyric acid accumulation in silage (Li et al., 2017). Supporting their assumption, we found that *Pantoea* was positively related to butyric acid content, and negatively related to lactic acid content. As a Gram-negative aerobic aerobe, *Sphingomonas* is commonly found in plants (White et al., 1996) and has ability to degrade various xenobiotic compounds such as herbicides and pesticides (He et al., 2017). Ogunade et al. (2018) found that the genus *Sphingomonas* was negatively correlated with ammonia-N content in alfalfa silage, and inferred this genus may be beneficial for protein preservation. In agreement with their reports, a negative correlation between *Sphingomonas* and NH_3_-N content was observed in this study. *Rhizobium* is usually distributed in the soil where legumes grow and is involved in N fixation by legumes (Zou et al., 2021).

Compared with the CK treatment, PL and LPL treatments upregulated the metabolism of membrane transport, carbohydrate, lipid, and terpenoids and polyketides. This might be due to the fact that the addition of LAB enhanced the utilization of available carbohydrates to produce lactic acid, acetic acid, and terpenoids and polyketides, which transported these fermentation substrates and end-products intracellular or extracellular for bacterial cell division (Kanehisa, 2019; Hisham et al., 2022). Herein, the relative abundance of amino acid metabolism was higher in the additive-treated silages than the CK silage, which was inconsistent with the finding of Bai et al. (2022), who reported that amino acid metabolism was predicted to be downregulated in well-preserved silages. This phenomenon is difficult to explain, but the upregulation of amino acid metabolism might be related to increased lactic acid content in the additive-treated silages, as lactic acid formation involves processes of amino acid decarboxylation and arginine deamination (Bai et al., 2021). The LA and LPL treatments upregulated the metabolism of membrane transport, amino acid, lipid, energy, and xenobiotics biodegradation and metabolism. It is inferred that the laccase inclusion could enhance the activity of bacterial biodegradation to remove xenobiotics, which might need to mobilize metabolism routes link to amino acid, lipid, and energy (He et al., 2021). The functions of replication and repair, translation, nucleotide metabolism, transcription, and genetic information processing were downregulated by the additive treatments. These decreased genetic functions in the additive-treated silages were probably due to lower pH inhibiting the undesirable microorganisms, and reflected a more stable bacterial community in these silages. Furthermore, the increased metabolism of terpenoids and polyketides in the additive-treated silages might indicate an increase in the synthesis of terpenoids and polyketides that are related to promotion of antimicrobial activities for inhibiting pathogen (Hisham et al., 2022). Promotion of antimicrobial activity against pathogens would be supported by downregulated infectious diseases metabolism with the additives compared with the CK. It is worth to note that caution should be given when interpreting functional profile from genomic sequence-prediction as they may differ from genetic constituent of microbial community. Therefore, a need exists for using more omics approaches, such as metabolomics and proteomics, to further investigate the functions of bacterial community in silage.

## Conclusion

The bioaugmentation of LA and PL enhanced homolactic fermentation and reduced fermentation loss of alfalfa silage, and consequently improved silage fermentation quality and nutrient preservation. The contents of lactic acid, true protein, and WSC were increased, and the contents of acetic acid, NH_3_-N, and ethanol were decreased by LA and PL bioaugmentation. The addition of LA and PL reduced the bacterial diversity of alfalfa silage, increased RA of *Lactobacillus*, and decreased RA of *Enterobacter* and *Klebsiella*. Metabolic pathways in the silage associated with activities of actively reproducing bacteria fermenting available sugars into organic acids, terpenoids and polyketides were enriched with the addition of additives. The combination of LA and PL synergistically improved the fermentation quality and nutrients preservation, and bacterial community of alfalfa silage, and can be a promising strategy for improving alfalfa silage quality.

## Data availability statement

The datasets presented in this study can be found in online repositories. The names of the repository/repositories and accession number(s) can be found in the article/Supplementary material.

## Author contributions

XB: conceptualization, methodology, and writing. HF: resource. GG: data curation and formal analysis. WH: resource and formal analysis. QHL: validation and funding acquisition. QX: investigation and visualization. CW: writing—reviewing and editing. QL: visualization. LC: supervision and funding acquisition. All authors contributed to the article and approved the submitted version.

## Funding

This work was supported by funds from Fundamental Research Program of Shanxi Province (201901D211370), Excellent Doctoral Research Startup Project of Shanxi Province (SXYBKY2019012), Science and Technology Innovation Project of Shanxi Agricultural University (2020BQ04), and Animal Husbandry Key Discipline construction program in “1331 project” of Shanxi Province.

## Conflict of interest

The authors declare that the research was conducted in the absence of any commercial or financial relationships that could be construed as a potential conflict of interest.

## Publisher’s note

All claims expressed in this article are solely those of the authors and do not necessarily represent those of their affiliated organizations, or those of the publisher, the editors and the reviewers. Any product that may be evaluated in this article, or claim that may be made by its manufacturer, is not guaranteed or endorsed by the publisher.

## References

[ref1] Albrecht K. A. Muck R. E. (1991). Proteolysis in ensiled forage legumes that vary in tannin concentration. Crop Sci. 31, 464–469. doi: 10.2135/cropsci1991.0011183X003100020048x

[ref2] Allen M. S. Bradford B. J. Oba M. (2009). Board-invited review: the hepatic oxidation theory of the control of feed intake and its application to ruminants. J. Anim. Sci. 87, 3317–3334. doi: 10.2527/jas.2009-1779, PMID: 19648500

[ref3] AOAC (1990). Official Methods of Analysis (15th Edn.). Association of Official Analytical Chemists, Arlington, VA.

[ref4] Bachmann M. Wensch-Dorendorf M. Kuhnitzsch C. Kleinsteuber S. Popp D. Thierbach A.. (2022). Changes in composition and diversity of epiphytic microorganisms on field pea seeds, partial crop peas, and whole crop peas during maturation and ensiling with or without lactic acid bacteria inoculant. Microbiol. Spectr. 10, e00953–e00922. doi: 10.1128/spectrum.00953-22 PMC9431205 35946942

[ref5] Bai J. Ding Z. Ke W. Xu D. Wang M. Huang W.. (2021). Different lactic acid bacteria and their combinations regulated the fermentation process of ensiled alfalfa: ensiling characteristics, dynamics of bacterial community and their functional shifts. Microb. Biotechnol. 14, 1171–1182. doi: 10.1111/1751-7915.13785, PMID: 33666350 PMC8085944

[ref6] Bai J. Franco M. Ding Z. Hao L. Ke W. Wang M.. (2022). Effect of bacillus amyloliquefaciens and Bacillus subtilis on fermentation, dynamics of bacterial community and their functional shifts of whole-plant corn silage. J. Anim. Sci. Biotechnol. 13, 1–14. doi: 10.1186/s40104-021-00649-0 34991716 PMC8739699

[ref7] Blajman J. E. Vinderola G. Paez R. B. Signorini M. L. (2020). The role of homofermentative and heterofermentative lactic acid bacteria for alfalfa silage: a meta-analysis. J. Agric. Sci. 158, 107–118. doi: 10.1017/S0021859620000386 30142700

[ref8] Borreani G. Tabacco E. Schmidt R. J. Holmes B. J. Muck R. E. (2018). Silage review: factors affecting dry matter and quality losses in silages. J. Dairy Sci. 101, 3952–3979. doi: 10.3168/jds.2017-13837, PMID: 29685272

[ref9] Broderick G. A. Grabber J. H. Muck R. E. Hymes-Fecht U. C. (2017). Replacing alfalfa silage with tannin-containing birdsfoot trefoil silage in total mixed rations for lactating dairy cows. J. Dairy Sci. 100, 3548–3562. doi: 10.3168/jds.2016-12073, PMID: 28259401

[ref10] Broderick G. A. Kang J. H. (1980). Automated simultaneous determination of ammonia and total amino acids in ruminal fluid and *in vitro* media. J. Dairy Sci. 63, 64–75. doi: 10.3168/jds.S0022-0302(80)82888-8, PMID: 7372898

[ref11] Cai Y. Benno Y. Ogawa M. Kumai S. (1999). Effect of applying lactic acid bacteria isolated from forage crops on fermentation characteristics and aerobic deterioration of silage. J. Dairy Sci. 82, 520–526. doi: 10.3168/jds.S0022-0302(99)75263-X, PMID: 10194670

[ref12] Chen L. Bao X. Guo G. Huo W. Xu Q. Wang C.. (2021). Treatment of alfalfa silage with tannin acid at different levels modulates ensiling characteristics, methane mitigation, ruminal fermentation patterns and microbiota. Anim. Feed Sci. Technol. 278:114997. doi: 10.1016/j.anifeedsci.2021.114997

[ref13] Chen Q. Marshall M. N. Geib S. M. Tien M. Richard T. L. (2012). Effects of laccase on lignin depolymerization and enzymatic hydrolysis of ensiled corn Stover. Bioresour. Technol. 117, 186–192. doi: 10.1016/j.biortech.2012.04.085, PMID: 22613895

[ref14] Contreras-Govea F. E. Muck R. E. Broderick G. A. Weimer P. J. (2013). *Lactobacillus plantarum* effects on silage fermentation and in vitro microbial yield. Anim. Feed Sci. Technol. 179, 61–68. doi: 10.1016/j.anifeedsci.2012.11.008

[ref15] Debnath R. Saha T. (2020). An insight into the production strategies and applications of the ligninolytic enzyme laccase from bacteria and fungi. Biocatal. Agric. Biotechnol. 26:101645. doi: 10.1016/j.bcab.2020.101645

[ref16] Dong Z. Li J. Wang S. Dong D. Shao T. (2022). Time of day for harvest affects the fermentation parameters, bacterial community, and metabolic characteristics of sorghum-Sudangrass hybrid silage. mSphere 7, e00168–e00122. doi: 10.1128/msphere.00168-22 PMC9429962 35862805

[ref17] Du H. S. Wang C. Wu Z. Z. Zhang G. W. Liu Q. Guo G.. (2019). Effects of rumen-protected folic acid and rumen-protected sodium selenite supplementation on lactation performance, nutrient digestion, ruminal fermentation and blood metabolites in dairy cows. J. Sci. Food Agric. 99, 5826–5833. doi: 10.1002/jsfa.9853, PMID: 31206694

[ref18] Fillat U. Martin-Sampedro R. Gonzalez Z. Ferrer A. N. A. Ibarra D. Eugenio M. E. (2017). Biobleaching of orange tree pruning cellulose pulp with xylanase and laccase mediator systems. Cellul. Chem. Technol. 51, 55–65.

[ref19] Filya I. Muck R. E. Contreras-Govea F. E. (2007). Inoculant effects on alfalfa silage: fermentation products and nutritive value. J. Dairy Sci. 90, 5108–5114. doi: 10.3168/jds.2006-877, PMID: 17954751

[ref1002] Gharechahi J. Kharazian Z. A. Sarikhan S. Jouzani G. S. Aghdasi M. Hosseini Salekdeh G. (2017). The dynamics of the bacterial communities developed in maize silage. Microb. Biotechnol. 10, 1663–1676. doi: 10.1111/1751-7915.12751, PMID: 28696065 PMC5658587

[ref20] Graf K. Ulrich A. Idler C. Klocke M. (2016). Bacterial community dynamics during ensiling of perennial ryegrass at two compaction levels monitored by terminal restriction fragment length polymorphism. J. Appl. Microbiol. 120, 1479–1491. doi: 10.1111/jam.13114, PMID: 26923533

[ref21] Grimont P. A. D. Grimont F. (2015). “Klebsiella” in Bergey’s Manual of Systematics of Archaea and Bacteria. eds. Trujillo M. E. Dedysh S. DeVos P. Hedlund B. Kämpfer P. Rainey F. A.. (New York, NY: American Cancer Society), 1–26.

[ref22] Guo L. Yao D. Li D. Lin Y. Bureenok S. Ni K.. (2020). Effects of lactic acid bacteria isolated from rumen fluid and feces of dairy cows on fermentation quality, microbial community, and *in vitro* digestibility of alfalfa silage. Front. Microbiol. 10:2998. doi: 10.3389/fmicb.2019.02998, PMID: 31998275 PMC6962301

[ref23] He L. Li S. Wang C. Chen X. Zhang Q. (2021). Effects of vanillic acid on dynamic fermentation parameter, nitrogen distribution, bacterial community and enzymatic hydrolysis of stylo silage. Front. Microbiol. 12:690801. doi: 10.3389/fmicb.2021.690801, PMID: 34512568 PMC8424185

[ref24] He W. J. Zhang L. Yi S. Y. Tang X. L. Yuan Q. S. Guo M. W.. (2017). An aldo-keto reductase is responsible for fusarium toxin-degrading activity in a soil *Sphingomonas* strain. Sci. Rep. 7:9549. doi: 10.1038/s41598-017-08799-w, PMID: 28842569 PMC5573404

[ref25] Hisham M. B. Hashim A. M. Mohd Hanafi N. Abdul Rahman N. Abdul Mutalib N. E. Tan C. K.. (2022). Bacterial communities associated with silage of different forage crops in Malaysian climate analysed using 16S amplicon metagenomics. Sci. Rep. 12, 1–17. doi: 10.1038/s41598-022-08819-4 35501317 PMC9061801

[ref26] Irawan A. Sofyan A. Ridwan R. Hassim H. A. Respati A. N. Wardani W. W.. (2021). Effects of different lactic acid bacteria groups and fibrolytic enzymes as additives on silage quality: a meta-analysis. Bioresour. Technol. Rep. 14:100654. doi: 10.1016/j.biteb.2021.100654

[ref27] Kanehisa M. (2019). Toward understanding the origin and evolution of cellular organisms. Protein Sci. 28, 1947–1951. doi: 10.1002/pro.3715, PMID: 31441146 PMC6798127

[ref28] Ke W. C. Ding W. R. Xu D. M. Ding L. M. Zhang P. Li F. D.. (2017). Effects of addition of malic or citric acids on fermentation quality and chemical characteristics of alfalfa silage. J. Dairy Sci. 100, 8958–8966. doi: 10.3168/jds.2017-12875 28918135

[ref29] Kung L. Jr. Shaver R. D. Grant R. J. Schmidt R. J. (2018). Silage review: interpretation of chemical, microbial, and organoleptic components of silages. J. Dairy Sci. 101, 4020–4033. doi: 10.3168/jds.2017-13909, PMID: 29685275

[ref1001] Langille M. G. I. Zaneveld J. Caporaso J. G. McDonald D. Knights D. Reyes J. A.. (2013). Predictive functional profiling of microbial communities using 16S rRNA marker gene sequences. Nat. Biotechnol. 31, 814–421. doi: 10.1038/nbt.2676, PMID: 23975157 PMC3819121

[ref30] Li L. Yuan Z. Sun Y. Kong X. Dong P. Zhang J. (2017). A reused method for molasses-processed wastewater: effect on silage quality and anaerobic digestion performance of *Pennisetum purpereum*. Bioresour. Technol. 241, 1003–1011. doi: 10.1016/j.biortech.2017.04.117, PMID: 28637158

[ref31] Licitra G. Hernandez T. M. Van Soest P. J. (1996). Standardization of procedures for nitrogen fractionation of ruminant feeds. Anim. Feed Sci. Technol. 57, 347–358. doi: 10.1016/0377-8401(95)00837-3

[ref32] Logue J. B. Stedmon C. A. Kellerman A. M. Nielsen N. J. Andersson A. F. Laudon H.. (2016). Experimental insights into the importance of aquatic bacterial community composition to the degradation of dissolved organic matter. ISME J. 10, 533–545. doi: 10.1038/ismej.2015.131, PMID: 26296065 PMC4817675

[ref33] McDonald P. Henderson A. R. Heron S. J. E. (1991). The Biochemistry of Silage, (2nd Edn.). Marlow: Chalcombe Publications.

[ref34] Miller G. L. (1959). Use of dinitrosalicylic acid reagent for determination of reducing sugar. Anal. Chem. 31, 426–428. doi: 10.1021/ac60147a030

[ref35] Muck R. E. Hintz R. W. (2003). Effects of breeding for quality on alfalfa ensilability. Trans. ASAE 46, 1305–1309. doi: 10.13031/2013.15439

[ref36] Nazar M. Xu L. Ullah M. W. Moradian J. M. Wang Y. Sethupathy S.. (2022). Biological delignification of rice straw using laccase from *bacillus ligniniphilus* L1 for bioethanol production: a clean approach for agro-biomass utilization. J. Clean. Prod. 360:132171. doi: 10.1016/j.jclepro.2022.132171

[ref37] Ogunade I. M. Jiang Y. Cervantes A. P. Kim D. H. Oliveira A. S. Vyas D.. (2018). Bacterial diversity and composition of alfalfa silage as analyzed by Illumina MiSeq sequencing: effects of *Escherichia coli* O157: H7 and silage additives. J. Dairy Sci. 101, 2048–2059. doi: 10.3168/jds.2017-12876, PMID: 29274960

[ref38] Pahlow G. Muck R. E. Driehuis F. Elferink S. J. W. H. O. Spoelstra S. F. (2003). Microbiology of ensiling. Microbiology 42, 31–93. doi: 10.2134/agronmonogr42.c2

[ref39] Pang H. Qin G. Tan Z. Li Z. Wang Y. Cai Y. (2011). Natural populations of lactic acid bacteria associated with silage fermentation as determined by phenotype, 16S ribosomal RNA and recA gene analysis. Syst. Appl. Microbiol. 34, 235–241. doi: 10.1016/j.syapm.2010.10.003, PMID: 21282025

[ref40] Polley H. W. Wilsey B. J. Derner J. D. (2007). Dominant species constrain effects of species diversity on temporal variability in biomass production of tallgrass prairie. Oikos 116, 2044–2052. doi: 10.1111/j.2007.0030-1299.16080.x

[ref41] Queiroz O. C. M. Ogunade I. M. Weinberg Z. Adesogan A. T. (2018). Silage review: foodborne pathogens in silage and their mitigation by silage additives. J. Dairy Sci. 101, 4132–4142. doi: 10.3168/jds.2017-13901, PMID: 29685282

[ref42] Rajak R. C. Banerjee R. (2018). An eco-friendly process integration for second generation bioethanol production from laccase delignified Kans grass. Energy Convers. Manag. 157, 364–371. doi: 10.1016/j.enconman.2017.11.060

[ref43] Sherpa K. C. Ghangrekar M. M. Banerjee R. (2018). A green and sustainable approach on statistical optimization of laccase mediated delignification of sugarcane tops for enhanced saccharification. J. Environ. Manag. 217, 700–709. doi: 10.1016/j.jenvman.2018.04.008, PMID: 29654973

[ref44] Su R. Ni K. Wang T. Yang X. Zhang J. Liu Y.. (2019). Effects of ferulic acid esterase-producing *lactobacillus fermentum* and cellulase additives on the fermentation quality and microbial community of alfalfa silage. PeerJ. 7:e7712. doi: 10.7717/peerj.7712, PMID: 31608168 PMC6788448

[ref45] Sun Y. Gong X. Wang Z. Huang C. Ma X. Wang M. (2019). Two-step pretreatment of corn Stover silage using non-ionic surfactant and ferric nitrate for enhancing sugar recovery and enzymatic digestibility of cellulose. Appl Biochem. Biotechnol. 189, 65–75. doi: 10.1007/s12010-019-02988-1, PMID: 30863987

[ref46] Tabacco E. Borreani G. Crovetto G. M. Galassi G. Colombo D. Cavallarin L. (2006). Effect of chestnut tannin on fermentation quality, proteolysis, and protein rumen degradability of alfalfa silage. J. Dairy Sci. 89, 4736–4746. doi: 10.3168/jds.S0022-0302(06)72523-1, PMID: 17106105

[ref47] Travaini R. Morales Otero M. D. Coca M. Da-Silva R. Bolado S. (2013). Sugarcane bagasse ozonolysis pretreatment: effect on enzymatic digestibility and inhibitory compound formation. Bioresour. Technol. 133, 332–339. doi: 10.1016/j.biortech.2013.01.133, PMID: 23434810

[ref48] Van Soest P. J. Robertson J. B. Lewis B. A. (1991). Methods for dietary fibre, neutral detergent fibre, and non-starch carbohydrates in relation to animal nutrition. J. Dairy Sci. 74, 3583–3597. doi: 10.3168/jds.S0022-0302(91)78551-2, PMID: 1660498

[ref49] White D. C. Sutton S. D. Ringelberg D. B. (1996). The genus *Sphingomonas*: physiology and ecology. Curr. Opini. Biotechnol. 7, 301–306. doi: 10.1016/S0958-1669(96)80034-6 8785434

[ref50] Xian Z. Wu J. Deng M. Wang M. Tian H. Liu D.. (2022). Effects of Cellulase and *Lactiplantibacillus plantarum* on the fermentation parameters, nutrients, and bacterial Community in *Cassia alata* silage. Front. Microbiol. 13:926065. doi: 10.3389/fmicb.2022.926065, PMID: 35875586 PMC9301268

[ref52] Zhao S. Yang F. Wang Y. Fan X. Feng C. Wang Y. (2021). Dynamics of fermentation parameters and bacterial community in high-moisture alfalfa silage with or without lactic acid bacteria. Microorganisms 9:1225. doi: 10.3390/microorganisms9061225, PMID: 34200084 PMC8226466

[ref53] Zi X. Li M. Chen Y. Lv R. Zhou H. Tang J. (2021). Effects of citric acid and *Lactobacillus plantarum* on silage quality and bacterial diversity of king grass silage. Front. Microbiol. 12:631096. doi: 10.3389/fmicb.2021.631096, PMID: 33717021 PMC7953137

[ref54] Zou X. Chen D. Lv H. Zhang Q. Zheng P. (2021). Effect of ellagic acid on fermentation quality and bacterial community of stylo silage. Fermentation 7:256. doi: 10.3390/fermentation7040256

